# Serum D-dimer level as a predictor of neurological functional prognosis in cases of head injuries caused by road traffic accidents

**DOI:** 10.1186/s12873-022-00613-9

**Published:** 2022-03-26

**Authors:** Masahiro Asami, Shinji Nakahara, Yasufumi Miyake, Jun Kanda, Takahiro Onuki, Akira Matsuno, Tetsuya Sakamoto

**Affiliations:** 1 Department of Neurosurgery, Advanced Critical Care Center, Teikyo University Hospital, 2-11-1 Kaga, Itabashi-ku, Tokyo, 173-8606 Japan; 2 Department of Emergency Medicine, Advanced Critical Care Center, Teikyo University Hospital, 2-11-1 Kaga, Itabashi-ku, Tokyo, 173-8606 Japan; 3 Department of Neurosurgery, Teikyo University Hospital, 2-11-1 Kaga, Itabashi-ku, Tokyo, 173-8606 Japan

**Keywords:** Neurological functional predictors, Serum D-dimer, Head injuries, Road traffic accidents

## Abstract

**Background:**

The number of traffic fatalities is declining in Japan; however, a large proportion of head injuries are still attributable to traffic accidents. Severe head trauma may cause progressive and devastating coagulopathy owing to exacerbated coagulation and fibrinolysis, which results in massive bleeding and poor patient outcomes. D-dimer is a fibrinolytic marker, which remarkably increases in severe coagulopathy due to the exacerbated fibrinolytic system. Because the degree of coagulopathy is associated with patient outcomes, the D-dimer level is a useful prognostic predictor in patients with head trauma. However, the usefulness of D-dimer in cases of head trauma caused by road traffic accidents remains inadequately explored. In this study, we investigated the relationship between D-dimer levels and outcomes in head injuries caused by traffic accidents.

**Methods:**

We extracted data on traffic injuries from Japan Neuro-Trauma Data Bank Project 2015, which is a prospective multicenter registry of head injuries. The analysis included 335 individuals with no missing data. The outcome variable was the score of the Glasgow Outcome Scale (GOS), a neurological outcome index. The participants were categorized into the favorable outcome (GOS score ≥ 4) and poor outcome (GOS score ≤ 3) groups. The serum D-dimer levels at the time of admission were divided into four categories at the quartiles, and the reference category was less than the first quartile (< 17.4 µg/mL). We performed a logistic regression analysis with GOS as the dependent variable and D-dimer as a predictor and performed a multivariate analysis that was adjusted for 10 physiological parameters.

**Results:**

In the univariate analysis, all groups with serum D-dimer values ≥ 17.4 μg/dL showed significantly poorer outcomes than those of the reference group. In the multivariate analysis, after adjusting for other factors, D-dimer levels ≥ 89.3 μg/dL were an independent predictor of poor outcome.

**Conclusion:**

After adjusting for physiological parameters, high serum D-dimer levels can be an independent factor for predicting neurological prognosis in head trauma caused by road traffic accidents.

## Background

Road traffic accident (RTA) casualties in Japan have been declining in recent years. According to the National Police Agency of Japan, the numbers of injured persons and fatalities attributable to RTAs in 2020 were 368,601 and 2,839, respectively; these were the lowest levels recorded ever since 1948 [1]. However, many lives are still lost in RTAs, and severe head injuries attributable to RTAs continue to occur frequently. Pedestrians and cyclists, who account for half of all traffic injury cases in Japan, are susceptible to severe head injuries. According to police data [2] from 2019, head injuries accounted for 50.7% and 53.6% of deaths among pedestrians and cyclists, respectively, and 25.8% and 38.2% of deaths among car occupants and motorcyclists, respectively. A study in Japan based on a nationwide trauma registry (Japan Trauma Data Bank) [3] reported that 48.0% and 49.0% of head injuries among injured pedestrians and cyclists, respectively, were severe with an Abbreviated Injury Scale (AIS) score of 3 or higher; these proportions were significantly higher than those of other road users.

One of the causes of fatal head injury is massive bleeding due to substantial coagulopathy [4, 5]. Hemostasis mechanisms function with a balance between a coagulation system (forming a thrombus to stop bleeding) and a fibrinolytic system (dissolving a thrombus that is no longer needed after tissue repair). However, in severe trauma, the fibrinolytic system becomes relatively excessive due to the exhaustion of coagulation factors and exacerbation of the fibrinolytic system. This may result in progressive and devastating coagulopathy. Therefore, bleeding control may become difficult due to the breakdown of the hemostatic mechanism. Although the pathogenesis of this type of coagulation/fibrinolysis disorder in severe head injury has not been well documented, it has been reported that there is a significant correlation between tissue factor levels and serum D-dimer levels due to brain parenchymal injury [6]. The brain parenchyma contains more tissue factor per unit weight than other tissues. Because of this higher tissue factor per unit weight, it is prone to prominent coagulation and associated hyperfibrinolysis, especially in severe head trauma [7]. Patients with moderate and severe head trauma develop progressive disorders of the coagulation and fibrinolysis systems. D-dimer, a product of clot degradation by the fibrinolytic system, is markedly elevated with increased fibrinolysis [6].

When not limited to the mechanism of injury, the degree of coagulopathy is associated with the prognosis of patients with head injuries, and increased D-dimer levels in the early stage of head injury are associated with poor prognosis, reflecting injury severity. D-dimer levels rise earlier after injury (usually within one hour) than other serum biomarkers of coagulation do [8]. Additionally, injured patients with higher D-dimer concentrations have a poorer level of consciousness at hospital arrival [8], a more progressive deterioration of consciousness after hospital arrival [9, 10], and a poorer neurological prognosis [8–10] than those with lower D-dimer concentrations. Furthermore, D-dimer values in combination with age and consciousness level can be used to predict mortality among injured patients [8]. The Japan Neurotrauma Data Bank (JNTDB) has information on mechanisms of injury in severe cases, with 49% of injuries due to traffic accidents and 43% due to falls or tumbles. As the severity of a case increases, the likelihood that the individual was in an RTA is also high [11]. For severe cases, intensive care management is required, and serum D-dimer levels are often elevated in patients under such intensive care, which makes it difficult to interpret blood coagulation and fibrinolysis system disorders due to head trauma alone. Therefore, to the best of our knowledge, there are no reports examining the relationship between D-dimer levels and the prognosis of head injuries caused by RTAs.

Therefore, in this study, we aimed to examine the association between D-dimer level at hospital arrival and neurological prognosis at discharge of patients with severe head injuries due to RTAs. We used data extracted from the JNTDB Project 2015, a Japanese nationwide level multicenter registry of severe head injuries, and adjusted for covariates associated with patient prognoses in the previous JNTDB Project 2009 using multivariate analysis [12].

## Methods

### Research design

This study was a prospective study using data registered in the JNTDB Project 2015, a multicenter case registration project at the national level in Japan. We aimed to investigate whether D-dimer levels at hospital arrival could be a predictor of neurological prognosis in patients with head injuries caused by RTAs. The study was performed in accordance with the ethical standards of the Declaration of Helsinki (1964) and its subsequent amendments. The study protocol was approved by the Teikyo University Medical Research Ethics Committee (approval number 15–044-2). Oral informed consent was obtained at hospital admission. The ethics committee also approved the oral informed consent from all participants.

### Japan Neurotrauma Data Bank (JNTDB) 2015

The JNTDB is a prospective multicenter case registration project started in 1997 by the Japanese Society of Neurotrauma for the purpose of epidemiological research on severe head trauma in Japan. Registration targets were patients of all ages with head injuries, defined as those with a consciousness level of Glasgow Coma Scale (GCS) score ≤ 8 at hospital arrival or within 48 h after injury, undergoing neurosurgical operations including insertion of an intracranial pressure sensor. Cardiac arrest patients without evident head injuries were excluded, although they met the consciousness criterion; only patients with evident head injuries were included. Four large-scale national surveys of the project have been conducted to date (1998, 2004, 2009, and 2015), and case registration has been carried out for two years each [13]. A new project is still underway.

### Study participants

**Fig. 1:**
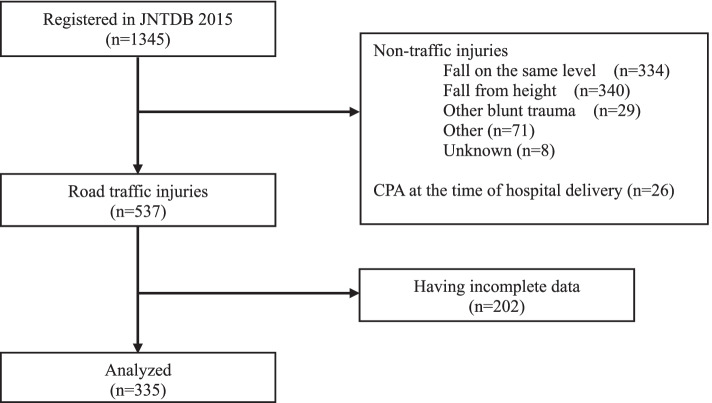
Selection of study participants. JNTDB: Japan Neurotrauma Data Bank

A total of 1345 patients were enrolled in the JNTDB Project 2015 from 32 facilities nationwide between April 1, 2015, and March 31, 2017. Of the 1345 registered cases, 782 non-traffic injuries and 26 cardiac arrest cases were excluded; the remainder (*n* = 537) were caused by vehicle collisions on the road. The analysis included 335 people, excluding those with missing data (Fig. 1).

### Outcome variable

The outcome variable was neurological function at hospital discharge, evaluated using the Glasgow Outcome Scale (GOS; Table 1). The JNTDB included data on GOS at discharge, evaluated and recorded by the doctor in charge. For the analysis, we divided the five classifications of GOS into two categories, a good prognosis (good recovery, moderate disability) and a poor prognosis (severe disability, persistent vegetative state, dead), and used them as a binary variable.

**Table 1:** Glasgow Outcome Scale (GOS) [14]

Score	Functional status	Clinical meaning
5	Good recovery	Light damage with minor neurological and psychological deficits
4	Moderate disability	No need for assistance in everyday life; employment is possible but may require special equipment
3	Severe disability	Severe injury with a permanent need for help with daily living
2	Persistent vegetative state	Severe damage with a prolonged state of unresponsiveness and a lack of higher mental functions
1	Death	Severe injury or death without recovery of consciousness

### Predictive variable

The main hypothetical predictive variable was the D-dimer level. The analysis included covariates to examine their relationship with the neurological prognosis and adjust for them to determine the independent association between D-dimer levels and the outcome. These covariates were factors that were found to be associated with prognoses in the past JNTDB projects and other factors that are likely to be associated with prognosis. The former factors included oxygenation, ventilation status, systolic blood pressure, body temperature, blood sugar, and multiple trauma, although they did not show consistent associations with patient prognoses in past projects (Table 2). The latter factors included demographic characteristics such as age and sex; physiological indicators such as respiratory rate; consciousness level; and overall injury severity indicated using the Injury Severity Score (ISS). The analysis did not include multiple trauma, defined in past JNTDB projects as having an AIS of 3 or higher trauma in other body areas in addition to the head, as a covariate to avoid multicollinearity in the multivariate analysis; because existence of multiple trauma was significantly associated with higher injury severity [12]. We did not include the insertion of an intracranial pressure monitor as a predictor because the performance of this procedure heavily depends on the capacities and policies of each facility in addition to the patients’ conditions.

**Table 2:** Comparison between previous projects [12]

Physiological parameters	P1998	P2004	P2009
Oxygenation	No relationship	No relationship	Worse hypoxia
Ventilation	Worse in hyperventilation	Slightly bad in hyper- and hypoventilation	No relationship
Systolic blood pressure	No relationship	Slightly bad in high and low blood pressures	Worse low blood pressure
Body temperature	_	No relationship, but in case of more than 39 °C, there is no good recovery or there is moderate disability	Worse hypothermia
Blood sugar	Worse in hyperglycemia	Worse in hyperglycemia	Worse in hyperglycemia
Multiple injuries	Worse in multiple injuries	No relationship	Worse in multiple injuries
ICP monitor	_	Worse in using ICP monitor	Worse in using ICP monitor

*ICP* intra cranial pressure

D-dimer level was not measured in these studies

All predictors were categorized. The categorizations of the consciousness level indicated as GCS score, respiratory rate, and systolic blood pressure followed those used in the Revised Trauma Score (an index to evaluate trauma severity based on physiological indicators) and were modified as needed. Categories with extremely small numbers were merged; a hypertension category was created in that of systolic blood pressure because hypertension may affect the prognosis of head trauma. The other variables used in the previous JNTDB projects followed the categorizations of those projects. Injury severity, indicated by the ISS, followed the categorization often used in previous studies. D-dimer categorization was made at quartiles because previous studies reported different cutoff values ​​for different prognostic indicators [7], and the higher the D-dimer value, the higher the probability of poor prognosis [6]. The variables were categorized as follows: 1) Serum D-dimer value at the time of hospital delivery were divided into four groups: < 17.4 μg/mL (reference category), ≥ 17.4 μg/mL and < 42.9 μg/mL, ≥ 42.9 μg/mL and < 89.3 μg/mL, and ≥ 89.3 μg/mL; 2) The sex of the patient was either male or female (reference category); 3) The ages of the patients were grouped as: < 15 years, 15 years to < 55 years (reference category), 55 years to < 65 years, and ≥ 65 years; 4) The patients’ levels of consciousness (GCS score) were categorized into five groups: 3, 4–5, 6–8, 9–12, and 13–15, where 15 indicated normal consciousness and 3 indicated deep coma; 5) A respiratory rate ≤ 9 times (three categories [0, 1–5, and 6–9 times] were combined into one to represent a decrease in respiratory rate) indicated hypoventilation, 10 to 29 times was normal, and ≥ 30 times indicated hyperventilation; 6) Arterial oxygen partial pressure (PaO_2_) < 60 mmHg indicated decreased oxygenation whereas PaO_2_ ≥ 60 mmHg was normal; 7) Arterial carbon dioxide partial pressure (PaCO_2_) < 35 mmHg indicated a hyperventilation state, PaCO_2_ ≥ 35 mmHg and < 45 mmHg was normal, and PaCO_2_ ≥ 45 mmHg indicated a hyperventilation state; 8) Systolic blood pressure (SBP) < 90 mmHg indicated a hypotensive state, SBP ≥ 90 mmHg and < 160 mmHg was normal, and SBP ≥ 160 mmHg indicated a hypertensive state; 9) Body temperature < 36℃ indicated hypothermia, ≥ 36℃ and < 37℃ was normal, and ≥ 37℃ indicated hyperthermia; 10) Blood glucose level < 200 mg/dL was normal and ≥ 200 mg/dL was indicative of hyperglycemia; and 11) To categorize injury severity, we combined two categories of the injury severity score (ISS) (< 15 and ≥ 16; < 25) into one (< 25) that was representative of low injury severity. Thus, a GCS score < 25 was the reference category, and a GCS score ≥ 25 indicated high injury severity.

### Analysis

Using a logistic regression model with neurological prognosis as the outcome, we analyzed the relationship between the outcome and D-dimer levels as well as the above-listed predictive variables. Normal values ​​were used as the reference category unless otherwise specified. Univariate and multivariate analyses were performed. In the multivariate analysis, we focused on the association between D-dimer levels and outcomes after adjusting for the other covariates. All variables were first input into the multivariate model, and then those with (odds ratios) ORs ≤ 1.5 or < 0.67 (the reciprocal of 1.5) were excluded to create the final model. We assumed that an odds ratio (effect size of this analysis) of 1.5 or higher was a meaningful contribution to the model.

The association between predictors and outcomes was evaluated using the odds ratio. The significance level was set at *p* < 0.05. Because this study used case registration data with a predetermined sample size, we calculated the minimum detectable effect size (OR) when comparing the ratios between the two groups. Assuming the sample size (*n* = 335), rate of high D-dimer levels (0.5), rate of poor prognosis when the D-dimer level was low (0.5), significance level (0.05), and power (0.8); the minimum odds ratio that achieved statistical significance was 1.87. IBM's SPSS Statistics 24 was used for statistical analysis, and G*Power 3.1.9.2 (Heinrich-Heine-Universität, Düsseldorf) [15] was used to calculate minimum detected OR. In addition, Cramer's V was performed to quantitatively examine the difference in characteristics between the study participants and the excluded participants; for Cramer's V, less than 0.2 was interpreted as no difference in characteristics [16].

## Results

### The participants’ attributes

Of the 335 people analyzed, the majority were male and vulnerable road users (pedestrians and cyclists; Table 3). The age distribution was bimodal, with peaks at approximately 20 and 70 years of age. The excluded cases due to missing data did not show apparent differences in characteristics from those included in the analysis (Tables 3 and 4).

**Table 3:** Characteristics of patients

	Analyzed cases (complete data) (*n *= 335)	Excluded cases (incomplete data) (*n* = 202)	Cramer’s V
Age, years, n (%)			
0 -14,	25 (7.5)	20 (9.9)	
15–24,	60 (17.9)	28 (13.9)	
25–34,	23 (6.9)	13 (6.4)	
35–44,	25 (7.5)	14 (6.9)	
45–54,	32 (9.6)	12 (5.9)	
55–64,	29 (8.7)	20 (9.9)	
65–74,	68 (20.3)	45 (22.3)	
75–84,	52 (15.5)	38 (18.8)	
85 ≤	21 (6.3)	12 (5.9)	0.101
Sex n, (%)			
Female	112 (33)	62 (31)	
Male	223 (67)	140 (69)	0.028
Road user type, n (%)			
Car	37 (11.0)	29 (13.4)	
Motorcycle	88 (26.3)	50 (23.0)	
Bicycle	69 (20.6)	53 (24.4)	
Pedestrian	141 (42.1)	69 (31.8)	
Unknown	0 (0.0)	1 (0.5)	0.112
Outcome			
Good	115(34.3)	74(36.6)	
Poor	220(65.7)	128(63.4)	0.023

**Table 4:** median and interquartile range of each parameter

	Analyzed cases (complete data) (*n* = 335)	Excluded cases (incomplete data) (*n *= 202)	Cramer’s V
D-dimer level (µg/mL), n			
17.4 >	83	21	
42.9 >	84	23	
89.3 >	84	11	
89.3 ≤	84	28	0.121
Glasgow coma scale, n			
13–15	41	36	
9–12	39	27	
6–8	151	69	
4–5	36	25	
3	68	45	0.116
Partial pressure of arterial oxygen(mmHg), n			
60 ≤	286	112	
60 >	49	10	0.085
Partial pressure of arterial carbon dioxide(mmHg)			
35–44.9	156	56	
35 >	68	28	
45 ≤	111	40	0.025
Systolic blood pressure(mmHg), n			
90–159	214	115	
90 >	31	25	
160 ≤	90	62	0.073
Body temperature (°C), n			
36–36.9	182	86	
36 >	101	42	
37 ≤	52	31	0.056
Blood sugar level(mg/dL), n			
200 >	244	115	
200 ≤	91	45	0.01
Injury Severity Score, n			
25 >	98	83	
25 ≤	237	88	0.19

### Factors related to neurological prognosis

Univariate logistic regression analysis showed that age ≥ 55 years, GCS score ≤ 8, respiratory rate ≤ 9 times, PaCO_2_ ≥ 45 mmHg, systolic blood pressure < 90 mmHg or ≥ 160 mmHg, body temperature < 36 °C, blood glucose level ≥ 200 mg/dL, D-dimer concentration ≥ 17.4 μg/mL, and ISS ≥ 25 were associated with poor prognosis (Table 5). In multiple logistic regression analysis, a D-dimer concentration ≥ 89.3 μg/dL was an independent predictor of poor prognosis (odds ratio, 7.37; 95% confidence interval, 2.03–26.86) after adjusting for the remaining covariates. Other independent predictors of poor prognosis included age ≥ 65 years, severe disturbance of consciousness (GCS score ≤ 8), and multiple trauma with an ISS ≥ 25.

**Table 5:** Results of logistic regression analyses to examine the associations between D-dimer levels and patient prognoses (*n* = 335)

		No. of patients	Patients with poor outcome (GOS≦3) *n*	cOR (95%CI)	aOR (95%CI)
D dimmer (μg/mL)					
	17.4 >	83	34	1.00	1.00
	42.9 >	84	48	1.92 (1.04–3.55)	1.35 (0.60–3.01)
	89.3 >	84	60	3.60 (1.89–6.86)	1.16(0.50–2.75)
	89.3 ≤	84	78	18.74 (7.33–47.89)	5.37 (1.72–16.73)
Sex					
	Female	112	79	1.00	___
	Male	223	141	0.72(0.44–1.17)	___
Age (years old)					
	65 ≤	141	126	9.15(4.88–17.18)	9.37 (4.16 -21.08)
	55–64	29	20	2.42(1.03–5.69)	1.83 (0.63 -5.35)
	15–54	140	67	1.00	1.00
	0–14	25	7	0.42(0.17–1.08)	0.33(0.10 -1.12)
Consciousness; Glasgow coma scale					
	13–15	41	12	1.00	1.00
	9–12	39	19	2.30(0.92–5.76)	1.13 (0.35–3.71)
	6–8	151	97	4.34(2.05–9.20)	2.56 (1.02–6.45)
	4–5	36	31	14.98(4.70–47.78)	9.29 (2.26 -38.12)
	3	68	61	21.06(7.51–59.09)	11.05 (3.12 -39.13)
Respiratory rate					
	10–29	275	179	1.00	___
	30 ≤	43	26	0.82(0.42–1.59)	___
	0–9	17	15	4.02(0.90–17.96)	___
Oxygenation PaO_2_ (mmHg)					
	60≦	286	187	1.00	1.00
	60 >	49	33	1.09(0.57–2.08)	1.68 (0.65 -4.32)
Ventilatory status PaCO_2_ (mmHg)					
	35–44.9	156	92	1.00	1.00
	35 >	68	47	1.56(0.85–2.85)	1.19 (0.51 -2.77)
	45≦	111	81	1.88(1.11–3.18)	1.80 (0.82 -3.93)
Systolic Blood pressure (mmHg)					
	90–159	214	120	1.00	1.00
	90 >	31	28	7.31(2.16–24.79)	2.43 (0.58 -10.26)
	160 ≤	90	72	3.13(1.75–5.61)	1.56 (0.73 -3.36)
Body temperature (°C)					
	36–36.9	182	110	1.00	1.00
	36 >	101	82	2.83(1.58–5.05)	1.60 (0.75 -3.42)
	37 ≤	52	28	0.76(0.41–1.42)	0.91 (0.39 -2.14)
Blood sugar (mg/dL)					
	200 >	244	152	1.00	ー
	200 ≤	91	68	1.79(1.04–3.07)	ー
Injury Severity Score					
	25 >	98	41	1.00	1.00
	25 ≤	237	179	4.29(2.61–7.07)	3.51 (1.77 -6.97])
H–L (*p*-value)					0.38

*IQR* Interquartile range, *cOR *Corrected odds ratio, *aOR *Adjusted odds ratio, *TBI *Traumatic brain injury, *OR *Odds ratio, *CI *Confidence interval, *H–L *Hosmer–Lemeshow test

## Discussion

This study found that high serum D-dimer levels in head injuries caused by RTAs could be an independent prognostic factor after adjusting for covariates. In the univariate analysis, the prognosis was significantly poorer in all categories with elevated serum D-dimer levels than in the reference category with the lowest value, and increased values were associated with an increased risk of poorer prognosis. In the multivariate analysis, the prognosis was significantly poor at high values ​​(≥ 89.3 µg/dL). In severe head trauma, the degree of blood coagulation/fibrinolytic injury affects bleeding control and is an important factor in determining prognosis [4, 5]. Our findings suggest that serum D-dimer level is a useful indicator for predicting poor prognoses of head trauma patients injured in RTAs as it identifies hyperfibrinolysis, which is the beginning of coagulopathy, at an early stage after injury.

The mechanism of coagulation and fibrinolysis in traumatic injuries, including traffic accidents, has not been elucidated, but conceptually, tissue injury releases tissue factors of the coagulation-fibrinolysis system, which induces systemic disseminated intravascular coagulation. It has been reported that damaged tissue factor can influence the extrinsic coagulation system, leading to an explosive increase in fibrinolysis. The brain tissue contains a large amount of tissue factor per unit weight, which is more pronounced in severe head trauma because brain parenchymal injury releases a large amount of tissue factor [7].

Furthermore, serum D-dimer levels are confirmed to rise within 1 h of head injury, and blood coagulation/fibrinolytic disorders are shown to spread across the whole body at the early stages of head injury [10]. In addition, among patients with severe head injury, the serum D-dimer level at hospital arrival was high in cases of exclusive head injury and polytrauma [17].

Our findings are consistent with several previous studies that reported associations between serum D-dimer levels and prognosis in patients with severe head injury, although the prognostic indicators varied in these studies, and there was a wide range of cutoff values. Takayama et al. [8] reported a 94.1% mortality rate among patients aged 57 years or older with a GCS score ≤ 7 and D-dimer level ≥ 50 µg/dL. Nakae et al. [10] reported that patients with both AIS ≥ 5 and D-dimer level > 37.5 µg/mL had a particularly high risk of progressive deterioration of consciousness and poor prognosis (Talk & Deteriorate) after hospital visit. Zhang et al. [9] examined 11 meta-analyses of traumatic brain injury and reported that the higher the D-dimer concentration value, the greater the increase in traumatic intracranial hematoma in eight of these meta-analyses. Although three meta-analyses showed no significant association for functional prognosis (GOS score ≤ 3) after 3 months due to study heterogeneity, a potential association remained.

In the multivariate analysis of this study, the dose–response relationship (higher serum D-dimer level was associated with a higher risk of poor prognoses) observed in the univariate analysis was not observed. Only at the highest level was the elevated D-dimer level a predictor of poor outcome. Serum D-dimer levels are often elevated in patients undergoing intensive care, which is difficult to interpret as the onset of coagulopathy due to head trauma alone. At lower levels of D-dimer, other factors may be more influential or predictive of prognosis. Specifically, higher age, deteriorated physiological status (indicated as impaired consciousness, slow respiratory rate, hypoventilation, hypertension, hypotension, hypothermia, and hyperglycemia), and severe injuries may have more influence on predictive abilities of poor outcomes than D-dimer values, which was reflected in the multivariate analysis.

In addition, the difference between the present and previous studies might have been influenced by the fact that various prognosis indices were used from study to study. This study used the GOS score at discharge, whereas previous studies used GOS scores 3 months after discharge or mortality within 3 months after injury. Utilization of long-term prognosis would increase the number of cases with poor prognosis. If the present study used a long-term prognosis indicator, lower serum D-dimer levels might have been associated with poor prognosis.

This study had several limitations. First, approximately 40% of the patients were excluded from the analysis due to missing values. The impact of this issue on the results is considered to be minimal because the comparison between the included and excluded patients showed no obvious difference in characteristics, and we assume that the defects are likely to occur randomly. The fact that there is no difference in the characteristics of the excluded and analyzed participants indicates that there is no correlation between the missing parameters and certain characteristics, and we considered that there is no selection bias. As a quantitative study, the Cramer’s V was calculated for Tables 3 and 4. The results were less than 0.2, and the difference was interpreted as small. Second, the timing of blood collection might have varied due to different treatment protocols by the hospital or different patient conditions (e.g., priority given to surgical treatment such as burr hole surgery in some facilities). The D-dimer values, which peak at 3 h after injury and then decline [8], may be underestimated or overestimated depending on the timing. Finally, a disparity in the calculation of the D-dimer value may exist between facilities depending on different test reagents used [18]. The Tokyo Clinical Laboratory Engineers Association [19] reported that the disparity in experiments using the same sample and the top three reagents, which account for 90% of the usage in Tokyo, was within ± 15% of the average value. At present, D-dimer values are not well standardized, but in this study, it is unlikely that the category will change significantly. Moreover, the effect on the results will be small because the values are divided into quartiles.

## Conclusion

Our study suggests that high serum D-dimer levels at hospital arrival can be an independent predictor of poor neurological prognosis at discharge in severe head injuries caused by RTAs. Future studies should examine the utility of D-dimer, based on more standardized measurements, to identify and manage coagulopathy at an early stage after injury to improve patient prognosis.

## Data Availability

The datasets generated and/or analyzed during the current study are not publicly available because of privacy/ethical restrictions but are available from the corresponding author on reasonable request.

## Abbreviations

AIS: Abbreviated injury scale
GCS: Glasgow Coma Scale
GOS: Glasgow Outcome Scale
ISS: Injury Severity Scale
OR: Odds ratio
PaCO_2_: Arterial carbon dioxide partial pressure
PaO_2_: Arterial oxygen partial pressure
RTA: Road traffic accident
SBP: Systolic blood pressure
